# Tissue diagnosis during colorectal cancer surgery using optical sensing: an in vivo study

**DOI:** 10.1186/s12967-019-2083-0

**Published:** 2019-10-02

**Authors:** E. J. M. Baltussen, S. G. Brouwer de Koning, J. Sanders, A. G. J. Aalbers, N. F. M. Kok, G. L. Beets, B. H. W. Hendriks, H. J. C. M. Sterenborg, K. F. D. Kuhlmann, T. J. M. Ruers

**Affiliations:** 1 Department of Surgery, Antoni van Leeuwenhoek Hospital – The Netherlands Cancer Institute, Amsterdam, The Netherlands; 2 Department of Pathology, Antoni van Leeuwenhoek Hospital – The Netherlands Cancer Institute, Amsterdam, The Netherlands; 3 Department of In-body Systems, Philips Research, Eindhoven, The Netherlands; 4 Department of Biomechanical Engineering, Delft University of Technology, Delft, The Netherlands; 5 Department of Biomedical Engineering and Physics, Amsterdam University Medical Centre, University of Amsterdam, Amsterdam, The Netherlands; 6 Faculty TNW, Group Nanobiophysics, Twente University, Enschede, The Netherlands

**Keywords:** Diffuse reflectance spectroscopy, Colorectal cancer, In vivo study, Supervised machine learning

## Abstract

**Background:**

In colorectal cancer surgery there is a delicate balance between complete removal of the tumor and sparing as much healthy tissue as possible. Especially in rectal cancer, intraoperative tissue recognition could be of great benefit in preventing positive resection margins and sparing as much healthy tissue as possible. To better guide the surgeon, we evaluated the accuracy of diffuse reflectance spectroscopy (DRS) for tissue characterization during colorectal cancer surgery and determined the added value of DRS when compared to clinical judgement.

**Methods:**

DRS spectra were obtained from fat, healthy colorectal wall and tumor tissue during colorectal cancer surgery and results were compared to histopathology examination of the measurement locations. All spectra were first normalized at 800 nm, thereafter two support vector machines (SVM) were trained using a tenfold cross-validation. With the first SVM fat was separated from healthy colorectal wall and tumor tissue, the second SVM distinguished healthy colorectal wall from tumor tissue.

**Results:**

Patients were included based on preoperative imaging, indicating advanced local stage colorectal cancer. Based on the measurement results of 32 patients, the classification resulted in a mean accuracy for fat, healthy colorectal wall and tumor of 0.92, 0.89 and 0.95 respectively. If the classification threshold was adjusted such that no false negatives were allowed, the percentage of false positive measurement locations by DRS was 25% compared to 69% by clinical judgement.

**Conclusion:**

This study shows the potential of DRS for the use of tissue classification during colorectal cancer surgery. Especially the low false positive rate obtained for a false negative rate of zero shows the added value for the surgeons.

*Trail registration* This trail was performed under approval from the internal review board committee (Dutch Trail Register NTR5315), registered on 04/13/2015, https://www.trialregister.nl/trial/5175.

## Background

Colorectal cancer is the third most common cancer worldwide [1]. Most tumors are located in the proximal colon (41%) followed by the rectum (28%) [2]. Surgery is the standard treatment, while patients with advanced rectal cancer are generally treated with a combination of neoadjuvant chemo- and radiotherapy [3]. In rectal cancer surgery there is a delicate balance between the complete removal of the tumor and sparing of vital surrounding tissue such as blood vessels, nerves and ureters. Damage to these structures leads to complications such as bladder and sexual dysfunction [4, 5]. In addition, the surgeon is confronted with limited space in the pelvic cavity as well as with fibrotic tissue induced by (chemo)radiotherapy. This often further impedes the determination of the exact tumor borders. These circumstances might lead to a positive resection margin (CRM), which is generally defined as tumor tissue within 2 mm from the resection surface. A positive CRM is a negative independent predictor of survival and local recurrence [6, 7]. Intraoperative tissue recognition could decrease the number of positive CRMs, while preventing complications that are caused by too extensive surgery. Currently there is no technique available which allows such intraoperative tissue type characterization in rectal cancer surgery.

**Fig. 1:**
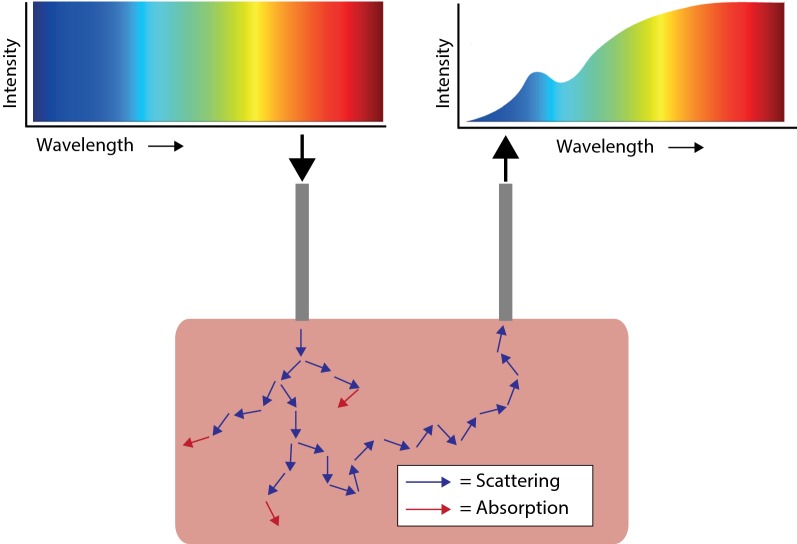
Basic principle of DRS. Light, with a broad wavelength range, is send through a fiber to the tissue. Within the tissue this light undergoes several interactions like scattering (blue arrows) and absorption (red arrows). Part of the light will be scattered to the surface where it is detected using a second fiber. The detected signal will be different than the signal that was send into the tissue due to the specific absorption of the tissue constituents. Based on the signal alterations different tissue types can be discriminated

Diffuse reflectance spectroscopy (DRS) might offer the possibility for intraoperative tissue recognition. In DRS, light from a broadband light source is sent into the tissue. In the tissue, the light undergoes several interactions such as scattering and absorption, before part of the light will be reflected back to the surface of the tissue. This light is collected and will form a spectrum which can be analyzed. The shape of the collected spectrum depends on the constituents of the tissue the light went through, which potentially allows discrimination of different tissue types (Fig. 1) [8]. DRS has already been successfully used in several different cancer types to distinguish between tumor and healthy tissue, e.g. in lung, breast, liver and head and neck cancer with accuracies of at least 77% [8–13].

So far, the use of DRS in colorectal cancer focused mainly on the application in colonoscopy [14–18]. These studies were performed in vivo and obtained sensitivities and specificities between 80–9 and 75–78%, respectively, for the detection of cancer tissue versus healthy tissue [16, 17]. The main difference between colonoscopy and a surgical setting is the fact that in colonoscopy tissue is assessed from inside the lumen, whereas in surgery the tissue is assessed from outside the lumen. Ex vivo studies focusing on DRS in colorectal surgery showed that tumor can be distinguished from healthy surrounding tissue with an accuracy of at least 91% [19–21].

This study investigates the role of DRS in colorectal cancer surgery in vivo. DRS measurement locations were determined by the surgeon and were located at the tumor and healthy surrounding tissues. The analysis of the measurements was done offline after surgery and was verified by pathological assessment. The aim of the study was to determine the accuracy of the DRS measurements in a surgical setting and to evaluate the added value when compared to the clinical judgement of the surgeon. Ultimately this could lead to a smart surgical tool for real-time peroperative tissue classification allowing more precise surgery.

## Materials and methods

### DRS system

**Fig. 2:**
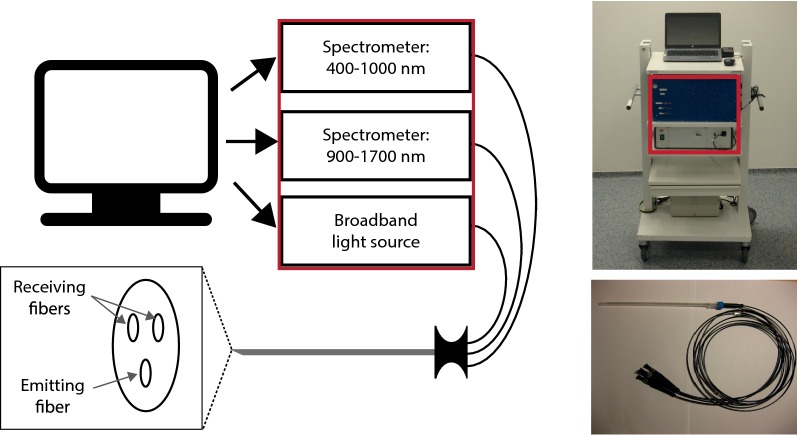
Measurement system. On the left a schematic image is shown of the system used to perform the measurements. The system consists of two spectrometers and a broadband light source, which are all controlled by a computer. Measurements are performed using a needle which includes three fibers. One that transports the light from the broadband light source to the tissue (emitting fiber) and two to transport the light from the tissue to the two spectrometers (receiving fibers). The distance between the receiving and emitting fibers is 1.29 mm. On the right, images are shown of the system as used during surgery (top image) and the needle used to perform the measurements with (bottom image)

The DRS system consists of two spectrometers, a Tungsten halogen broadband light source and an embedded shutter. The light source covers the visual and infrared wavelength range from 360 to 2500 nm. The two spectrometers cover most of this wavelength range as well, with one covering the visual wavelengths, 400 to 1100 nm, (Andor Technology, DU420ABRDD). The other spectrometer covers the near-infrared wavelength range of 900–1700 nm (Andor Technology, DU492A-1.7) (Fig. 2). Custom made LabView software (National Instruments, Austin, Texas) makes it possible to control the system and to acquire and save the data. A detailed description about calibration of the system can be found elsewhere [22, 23].

The measurements were performed using clinical-grade disposable 16 G needles (INVIVO, Gainesville, Florida). In the needles, three optic fibers (core diameter = 200 µm) were embedded: one fiber to illuminate the tissue and two fibers to transport the light from the tissue to the two spectrometers. The center to center distances between the emitting and receiving fibers was 1.29 mm. The distance between the emitting and receiving fibers determines the measurement depth, which is approximately the same as the distance between the fibers [24].

### Study protocol

This in vivo study was performed under approval from the internal review board of The Netherlands Cancer Institute (Dutch Trail Register NTR5315). Patients from the Netherlands Cancer Institute, were included, based on preoperative imaging. Patients were selected for inclusion when preoperative imaging indicated colorectal cancer stage cT3 or cT4, and patients would undergo open surgery for tumor removal. All included patients signed informed consent. All ethical guidelines were followed.

During surgery, the surgeon was asked to acquire DRS measurements of healthy fat, healthy colorectal wall and tumor tissue. To obtain the spectra, a needle was placed by the surgeon in contact with the tissue. For tumor measurements, three locations were measured on the surface of the bowel wall which were classified by the surgeon as most suspect for tumor. The surgeons were asked to indicate how certain he or she was that these measurements contained tumor, which was noted. Fat and healthy colorectal wall were measured at a distance from the tumor, to ensure these locations were actually healthy. Per location, the measurement was repeated three times. All locations were marked with a suture. If the surgeon was unable to localize tumor close to or at the surface of the bowel wall no measurements were performed and the patient was excluded from any further analysis. After surgery, the sutures marking the measurement locations were removed and replaced by ink which was visible during microscopic inspection. Subsequently, the specimen was brought to the pathology department and was further processed according to standard protocol. All measured and marked locations were included in hematoxylin-eosin (H&E) coupes.

### Pathology classification

Histopathological validation of the DRS measurements was performed by an experienced colorectal pathologist. To this end, the H&E coupes were examined, under a microscope, and the different tissue types observed in the H&E coupe were labeled as fat, healthy colorectal wall or tumor. Subsequently, the labeled tissue types were correlated to the DRS measurements which on their turn were classified as as fat, healthy colorectal wall or tumor measurements. A measurement was classified as tumor when tumor was present within 1.5 mm from the surface.

For some tumor measurements correlation with histopathology analysis was inconclusive. Correlation with histopathology was classified as inconclusive if tumor was present on the H&E coupe over a length of less than 0.5 mm, within 1.5 mm from the measurement surface. For such small tumor areas correlation with histopathology was too inaccurate to conclude whether tumor was measured or not. To reduce the influence of these measurements on the classification, these were removed from the dataset.

### Data analysis

**Fig. 3:**
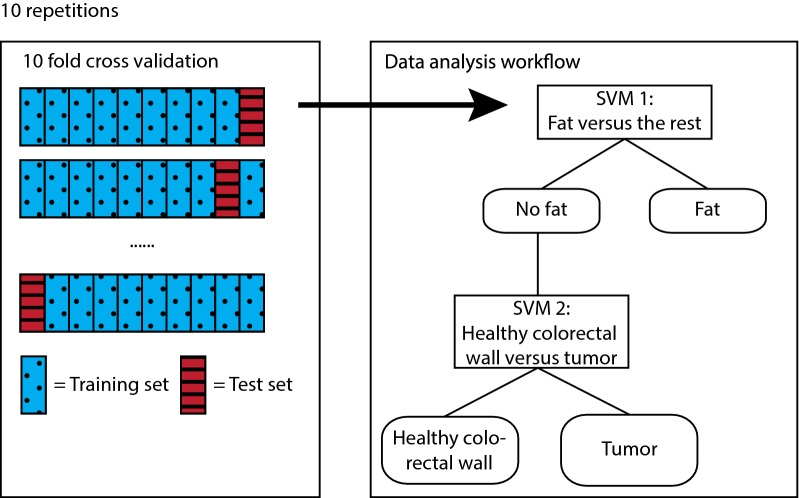
Data analysis workflow

Data analysis was performed using Matlab (version 8.5, MathWorks Inc., Natick, Massachusetts). First, all spectra were calibrated using a white reference and dark reference taken before the measurements of each patient [25]. Before classification, all spectra were normalized at 800 nm. Using the entire spectra, two linear support vector machines (SVM) were trained using a tenfold cross-validation to distinguish the three tissue types; fat, healthy colorectal wall and tumor. An SVM is a machine learning technique and a binary classifier, able to distinguish two different classes at once. The first SVM was a one versus all classification to distinguish fat from healthy colorectal wall and tumor. The second SVM was used to separate healthy colorectal wall from tumor. For the training of the first SVM, healthy colorectal wall and tumor were merged into one class. The training dataset of the second SVM only included healthy colorectal wall and tumor spectra. For testing of both SVMs the result of the first SVM determined whether the spectrum was given as an input to the second SVM. Spectra that were not classified as fat were also classified by the second SVM to distinguish between healthy colorectal wall and tumor tissue (Fig. 3). The tenfold cross-validated training and testing of both SVMs was repeated ten times to ensure representative results.

To evaluate the classification results the Matthews Correlation Coefficient (MCC) (Eq. 1) was used together with the accuracy, sensitivity and specificity. The MCC was used because it is less influenced by imbalanced data compared to the accuracy value. The MCC gives a value between − 1 and 1, where − 1 stands for complete reverse classification by the classifier, + 1 for a perfect classification by the classifier and 0 for no better than random classification by the classifier. In Eq. 1 TN, TP, FN and FP are the number of true negatives, true positives, false negatives and false positives respectively.

1 $$MCC = \frac{TP \times TN - FP \times FN}{{\sqrt {\left( {TP + FP} \right)\left( {TP + FN} \right)\left( {TN + FP} \right)\left( {TN + FN} \right)} }}$$

### Depth analysis

**Fig. 4:**
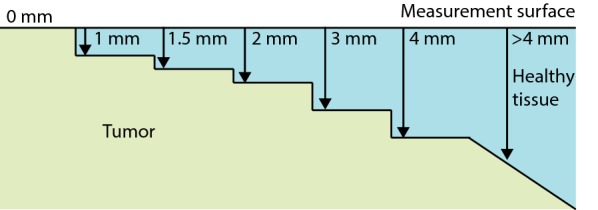
Maximum distance from measurement surface to tumor for a measurement to be classified as tumor

With a distance of 1.29 mm between the emitting and receiving fibers, tumor could be detected up to 1–1.5 mm in depth [26]. Therefore, measurements were classified as tumor when tumor was present within 1.5 mm from the measure surface. The influence of the depth of the tumor on the classification result was analyzed as well. This was done by increasing the maximum distance, for a measurement to be classified as tumor, between the measurement surface and the first encountered tumor tissue (Fig. 4). The distance was increased from 0 mm to more than 4 mm. For each distance the classification was re-trained and re-tested and accuracies and MCC values were obtained.

### Comparison to clinical judgement

To determine the added value of the DRS technique to the clinical judgement of the surgeon, results of the classification of the DRS measurements were compared to the tissue classification given by the surgeon. Most added value is obtained on locations of which the surgeon is uncertain whether tumor is present or not. Therefore, locations of which the surgeon indicated not to be sure about the presence of tumor were evaluated separately. To avoid positive resection margins the number of false negative classified locations, the number of locations classified as healthy tissue that were actually tumor, should be zero. To avoid false negative classifications, a new threshold for the classifier to classify a location as tumor had to be determined. For objective evaluation of the uncertain locations, this threshold was determined based on the locations of which the surgeon was certain. Thereafter, this threshold was applied to the classification of the uncertain locations and the results were compared to the judgement of the surgeons.

## Results

### Inclusion

In total, 52 patients were included in the study. Patient and tumor characteristics of the included and measured patients are described in Table 1. Eventually, 20 patients were not measured. Four patients were not measured because during surgery, the surgeon was not able to visualize tumor at the bowel surface, of which 2 patients were staged pT4, 1 pT3 and 1 pT0. The other 15 patients were not measured because of logistical reasons. Logistical issues included; surgery that was performed in another hospital, theatre time did not allow additional time for measurements, patients had too extensive disease because of which no resection was performed and therefore no pathology evaluation of the measurement was possible, and changes in the operation room schedule. Of the included patients with rectal cancer, four received neoadjuvant radiotherapy, three in combination with chemotherapy. One patient received neoadjuvant chemotherapy only.

**Table 1:** Patient and tumor characteristics

	Included	Measured
Total number of patients	52	32
Gender		
Male	29	19
Female	23	13
Age		
Median	59	61
Interquartile range	50–68	50–68
Tumor location		
Appendix	1	0
Cecum	7	2
Colon	24	17
Sigmoid	13	8
Rectum	7	5
Stage after histopathology^a^ evaluation		
pT0	2	1
pT1	0	0
pT2	2	2
pT3	22	14
pT4	24	13
Recurrence	2	2
Exclusion		
No tumor at surface	4	–
Surgery at another hospital	1	–
Theater time	2	–
Too extensive disease	6	–
Changes in schedule	7	–

^a^T stages include staging after pathological evaluation

**Fig. 5:**
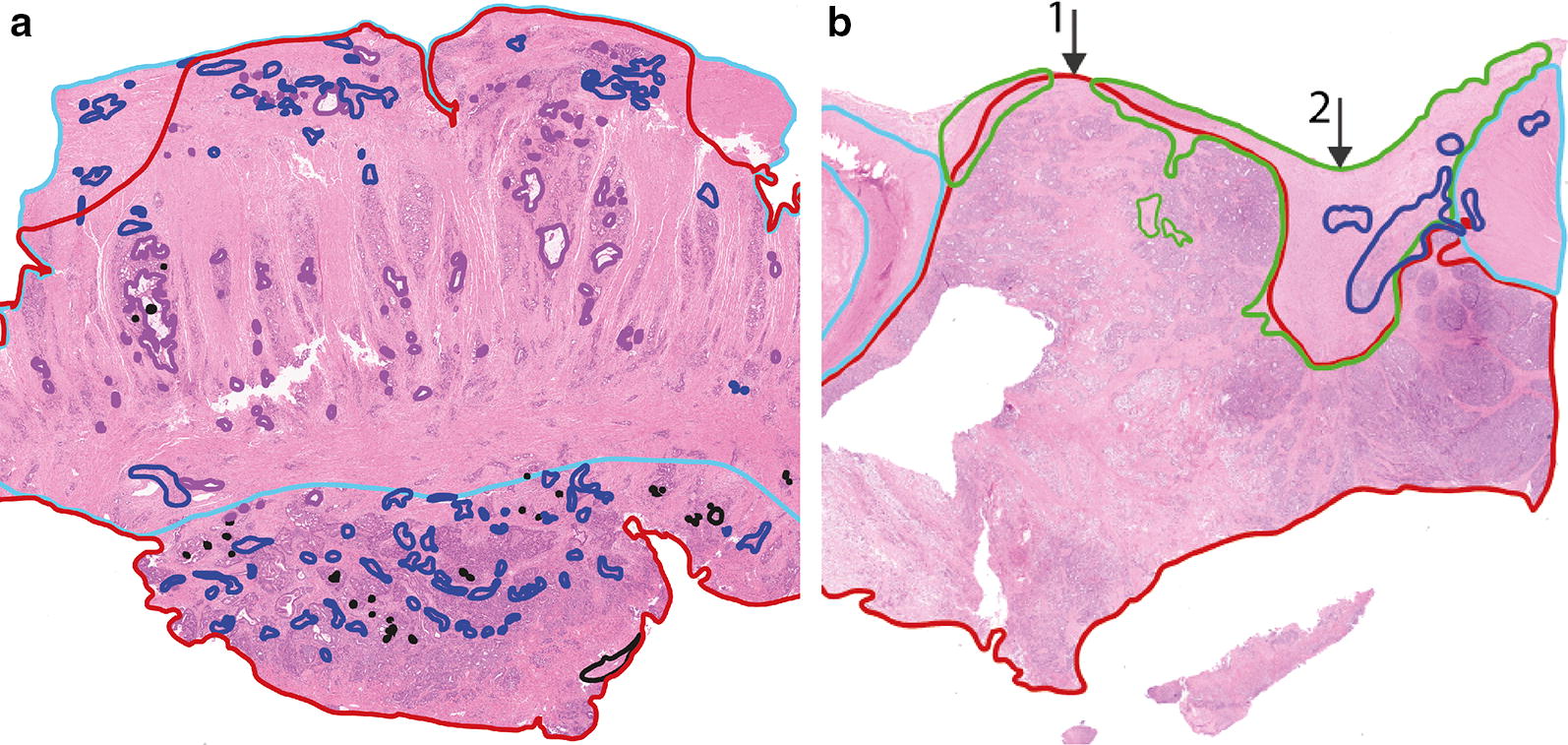
H&E slides of a measured locations with conclusive and inconclusive correlation to histopathology. H&E slides were annotated by a pathologist. Red = tumor, light blue = muscle, green = fibrosis, dark blue = inflammation. **a** Conclusive histopathology, with a large area of only tumor at the surface. **b** Inconclusive histopathology, if the measurement would have been on location 1, it would be a tumor measurement, however on location 2, less than 0.5 mm to the right it would be a fibrosis measurement. Locations with histopathology similar to **b** were excluded whereas locations with histopathology similar to **a** were used for classification

**Fig. 6:**
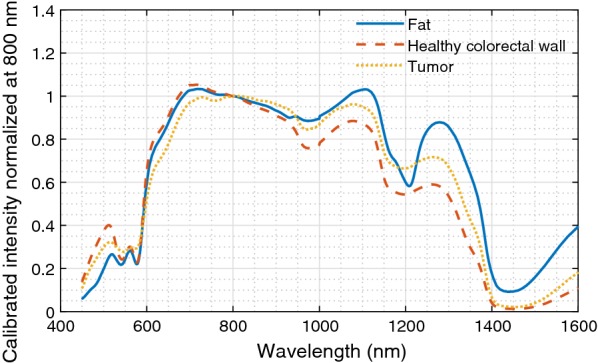
Mean spectra of fat, healthy colorectal wall and tumor, normalized at 800 nm

In total, 835 spectra (from 270 locations) were acquired, 402 on fat, 282 on healthy colorectal wall, and 151 on tumor. Histopathology was not available for the tumor locations of one of the patients, therefore 9 spectra were excluded for further analysis. After removal of all tumor measurements with inconclusive histopathology (Fig. 5b), 87 tumor measurements were left. For fat and healthy colorectal wall, most measured locations consisted entirely of fat or healthy colorectal wall, respectively. In Fig. 6 the mean spectra of fat, healthy colorectal wall and tumor are shown, the spectra are normalized at 800 nm.

### Classification results

**Fig. 7:**
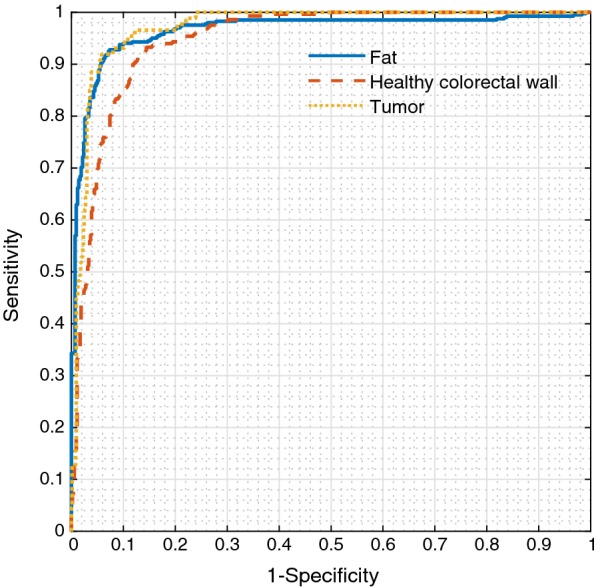
ROC curves of one iteration for all three tissue types

The evaluation of the classification was performed per tissue type, all values were calculated as a one versus all classification. Classification of fat was done with a mean MCC of 0.83, classification healthy colorectal wall with a mean MCC of 0.77 and tumor with a mean MCC of 0.73. In Table 2 the mean accuracy, MCC, sensitivity and specificity values are shown for all tissue types. In Fig. 7 the ROC curves of each tissue type are shown. For each tissue type, one iteration of the ten repetitions is shown. The average accuracy over all tissue types, weighted based on the number of measurements per tissue type, was 0.91.

**Table 2:** Mean values (STD) of accuracy, MCC, sensitivity and specificity, per tissue type

Tissue type	Accuracy	MCC	Sensitivity	Specificity
Fat	0.92 (0.00)	0.83 (0.01)	0.89 (0.01)	0.94 (0.00)
Healthy colorectal wall	0.89 (0.01)	0.77 (0.01)	0.92 (0.01)	0.87 (0.01)
Tumor	0.94 (0.00)	0.73 (0.02)	0.90 (0.02)	0.94 (0.00)

### Depth analysis

**Fig. 8:**
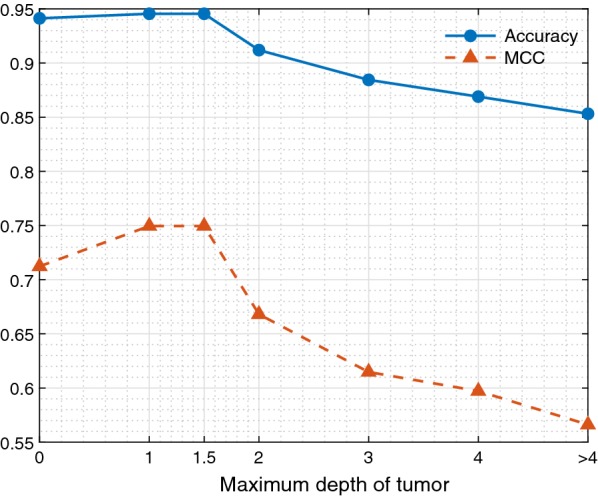
The accuracy and MCC values for tumor tissue. With increasing maximum depth for tumor measurements to be classified as tumor

To examine the influence of the depth of the tumor, the distance between the measurement surface and the first encountered tumor tissue, was varied. The first step was to include only measurements with tumor at the surface, so at 0 mm in depth. From this a 1 mm increase in depth was taken. The depth of 1.5 mm was included as well, because this distance was used in the original analysis. In Fig. 8 the resulting accuracies and MCC values for the different depth are shown for tumor. Both the accuracy and MCC show an optimum around 1–1.5 mm. Accuracy and MCC decrease if the tumor starts at a depth of 2 mm or more.

### Comparison to clinical judgement

The surgeon indicated not to be sure whether tumor was measured in 54 out of 270 locations. For these locations the technique could be of added value by providing the surgeon with more information about the tissue type. For the analysis of these locations, the threshold of the classifier was adjusted such that no false negative classifications were obtained on the locations of which the surgeon was certain. With this adjusted threshold, the uncertain locations were classified. The classification of the uncertain locations resulted in 25% of the healthy locations falsely classified as tumor and no measurements on tumor tissue classified as healthy tissue. When evaluating the judgement of the surgeons, 69% of the healthy tissue locations were incorrectly classified as tumor by the surgeon. In Table 3, an overview is given on the classification results of the classifier and surgeon compared to the histopathology. Locations are separated between healthy and tumor, where healthy included fat and healthy colorectal wall.

**Table 3:** Confusion matrix of histopathology classification and judgement by the surgeon and the classification by the classifier of the 54 measurement locations of which the surgeon was uncertain

	Classification by surgeon		Classification by classifier	
	Healthy	Tumor	Healthy	Tumor
Histopathology				
Healthy	16 (31%)	36 (69%)	39 (75%)	13 (25%)
Tumor	0 (0%)	2 (100%)	0 (0%)	2 (100%)

## Discussion

To the best of our knowledge this is the first in vivo study using DRS to distinguish tumor tissue from healthy surrounding tissues in colorectal cancer surgery. It is shown that tumor tissue can be distinguished from healthy colorectal wall and fat with a sensitivity and specificity of 0.90 and 0.94, respectively, giving an accuracy of 0.94.

Previous studies using DRS to discriminate colorectal tumor tissue from healthy surrounding tissue were mainly focused on the application during endoscopy [14–18] or were performed ex vivo [19–21]. The endoscopy studies showed a major difference in blood content between tumor and healthy mucosal tissue. In these studies, only visible wavelengths were included in the analysis. As blood is the main absorber in this wavelength range, differences in blood content can reliably be determined. In the current study differentiation between tumor and healthy tissue needs to be made during surgery, were the presence of blood on the measurement surface cannot always be controlled. This makes parameters obtained in the wavelength region of blood absorption less reliable for classification. For this reason, also the near-infrared wavelength range was included, to be able to obtain additional parameters outside of the blood absorption wavelength range [14–18]. Moreover, during endoscopy healthy surrounding tissue only consists of mucosal tissue from the lumen of the colon. During surgery mucosal tissue will not be encountered, but fat and bowel muscle tissue will be. Therefore, during the surgical application of DRS, tumor has to be differentiated from fat and muscle tissue, instead of from mucosal tissue like in colonoscopy. Discrimination between fat and tumor tissue seems an easy task [20]. As shown by the MCC values in Table 2 the separation of healthy colorectal wall and tumor tissue is more difficult.

When comparing the current study to the results obtained previously in the ex vivo studies, the accuracies seem similar, ranging from 91 to 99% [19–21]. If the imbalance in the current in vivo dataset is taken into account and a weighted average is taken for all three tissue types, an accuracy of 0.91 for the current in vivo study is obtained. In the ex vivo study an average accuracy over all tissue types of 0.95 was found, which is slightly higher [20]. The main reason for the difference in accuracies is the less controlled measurement environment for the current in vivo study. This will lead to less accurate correlation with histopathology for the evaluation of the classification, which will lead to a decrease in accuracy. In Table 2 the results are shown for the classification in which only conclusive histopathology was included. If all measured locations are included, including the ones of which histopathology classification was inconclusive (Fig. 5b), the MCC values of healthy colorectal wall and tumor show a decrease to 0.67 and 0.56 respectively. Which indeed shows that uncertainty in the histopathology correlation will influence the outcome of the classification. This problem is hard to circumvent. One way to get a more reliable accuracy on the differentiation between healthy colorectal wall and tumor is to increase the number of included patients. With an increase in the number of patients, at least the number of patients with clear histopathology will increase and potentially also the ratio with the number of patients with unclear histopathology.

Furthermore, during the ex vivo studies it is simple to obtain measurement locations with pure tissue types. In the current study this was not always possible, because tumor did not always penetrate the bowel wall. Therefore, some of the tumor measurements were performed with a small layer of healthy colorectal wall between the measurement surface and tumor. If the maximum depth of tumor from the measurement surface was increased from 0 up to more than 4 mm, a drop in accuracy and MCC value for tumor is shown for depth of tumor more than 1.5 mm (Fig. 8). This is most likely due to the small amount or absence of tumor present in the measured volume. Therefore, classification of these measurements is harder or even impossible. The measurement volume is mainly determined by the distance between the emitting and receiving fibers. In the current study the fibers were 1.29 mm apart resulting in a measurement depth of approximately 1–1.5 mm. If this distance is increased the measurement volume will increase and with this the depth until which tumor can be detected. Therefore, with an increase in distance between the receiving and emitting fibers, the accuracy of tumor detection at larger depths will be better. The decrease in accuracy and MCC value for measurements with tumor at the surface (0 mm), is most likely due to the low number of measurements in this group.

Since clinically a tumor free margin (CRM) is defined as > 2 mm, correct classification of measurements with a maximum depth of 2 mm will be more useful for the surgeon than a classification which includes also tumor tissue deeper than 2 mm. The current technique will provide an average of the tissue types in the entire measurement volume. Therefore, if the volume is up to 2 mm in depth, the surgeon can act on the information provided by the technique, because if tumor is indicated by the technique, tumor will be present within 2 mm from the resection margin, resulting in a positive CRM. Whereas, if it would provide information from further than 2 mm in depth it would be hard for the surgeon to determine whether to act on it or not. Since the current technique cannot locate the depth of the tumor, tumor could still be more than 2 mm from the resected surface, resulting in a negative CRM, but it could also be within 2 mm from the resected surface where it will cause a positive CRM.

In this study four patients were excluded because the surgeon indicated that no tumor could be measured. No measurements were performed in these patients to ensure a sufficient tumor to healthy measurement ratio for further classification. Of these four excluded patients, two tumors were staged by pathology as pT4, one as pT3 and one as pT0. The patients with pT4 staged tumors received neoadjuvant chemotherapy and showed a significant inflammatory reaction around the tumor area. It would have been possible to measure tumor at the surface or close to the surface of the bowel wall of these two patients. However, the surgeon was unable to distinguish tumor from inflammation. Therefore, the surgeon performed a more extensive resection to prevent positive margins. Due to the more extensive surgery, the surgeon was unable to perform measurement close to the tumor. These typical cases, where the surgeon was unable to discriminate tumor tissue from healthy tissue and therefore extended the resection, illustrate once again the need for a technique that can real-time classify tissue during surgery.

When the threshold of the classification is set such that the classification will not give any false negative predictions, the added value of the technique is shown for the uncertain locations (Table 3). For these locations the surgeon indicated not to be sure whether there was tumor present or not. We defined our threshold such that no false negative predictions were allowed, so no tumor locations should be classified as healthy, avoiding positive resection margins. As shown in Table 3 the DRS technique causes a large decrease in the number false positive classified locations compared to clinical judgement. Classification of the DRS measurements resulted in 25% of the locations wrongly classified as tumor, instead of 69% of the locations wrongly indicated as tumor by the surgeon. The specificity of 75% (Table 3) is lower than the 0.94 as shown in Table 2, this is due to defining the sensitivity at 1.00, which inevitably results in a decrease of the specificity. However, even by setting the sensitivity at 1.00 we observed a significant decrease in false positive classified locations with DRS compared to the surgeon. From these results is can be concluded that DRS can potentially result in less extensive surgery and thereby might lead to less complications during and after surgery.

The translation of DRS into clinical practice has taken some time. With this study a major step is taken to introduce DRS in the clinic. This study shows that it is possible to use DRS during colorectal cancer surgery to discriminate healthy tissue from tumor tissue. Further development of the technology could lead to incorporation of the technology in a smart surgical tool. When the technique is used in the form of a hyperspectral camera it is also possible to incorporate it into a laparoscopic camera or during robotic surgery.

## Conclusion

In this in vivo study, tumor can be classified with an accuracy of 0.94 and a sensitivity and specificity of 0.90 and 0.94, respectively. The false positive rates from the judgement of the surgeon and the false positive rate of the classification of the DRS spectra, demonstrate the potential of using DRS in vivo in colorectal cancer. Future research should focus on making the analysis of the obtained spectra real-time, to enable in vivo evaluation of the technology.

## Data Availability

The dataset generated and analyzed in this research are not publicly available because of privacy reasons. Upon reasonable request the corresponding author can make them available.

## Abbreviations

DRS: diffuse reflectance spectroscopy
H&E: hematoxylin–eosin
SVM: support vector machine
CRM: circumferential resection margin
MCC: Matthews Correlation Coefficient
STD: standard deviation
ROC: receiver operating curve
